# Supplementary material for: Causal interplay between lactose intolerance and gut microbiota: a combined bidirectional Mendelian randomization and in vivo validation study
Source: Front Nutr. 2026 Jun 1;13:1803337. doi: 10.3389/fnut.2026.1803337 (PMC13265576; doi:10.3389/fnut.2026.1803337)
Supplement: Supplementary file 1 [file Data_Sheet_1.zip › supplementary materials/Forward/forest plot/ebi-a-GCST90027701.finngen_R12_E4_LACTONAS.pdf]

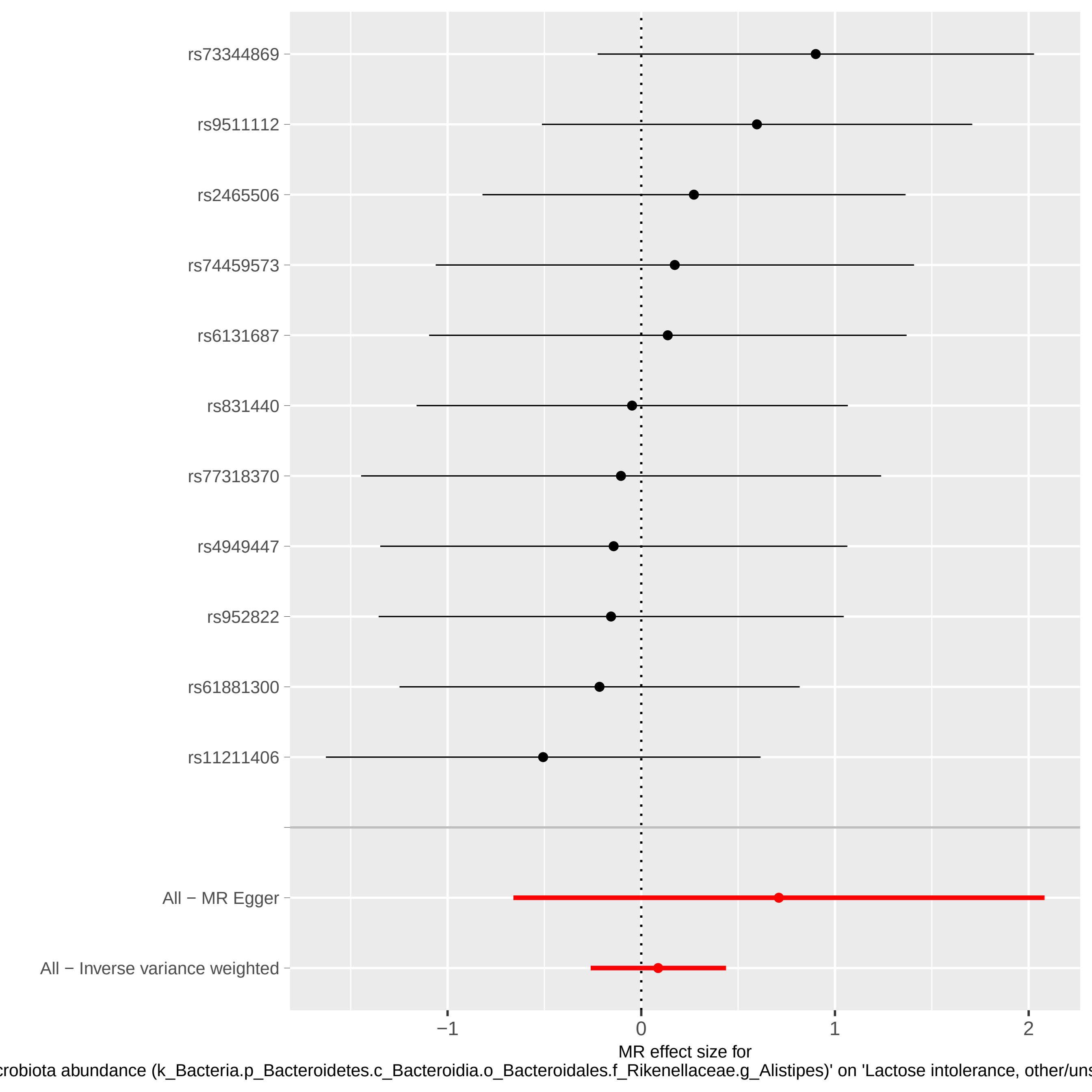
rs73344869
rs9511112
rs2465506
rs74459573
rs6131687
rs831440
rs77318370
rs4949447
rs952822
rs61881300
rs11211406
All − MR Egger
All − Inverse variance weighted
−1
0
1
2
MR effect size for
crobiota abundance (k_Bacteria.p_Bacteroidetes.c_Bacteroidia.o_Bacteroidales.f_Rikenellaceae.g_Alistipes)' on 'Lactose intolerance, other/uns
